# Molecular analysis of the diversity of vaginal microbiota associated with bacterial vaginosis

**DOI:** 10.1186/1471-2164-11-488

**Published:** 2010-09-07

**Authors:** Zongxin Ling, Jianming Kong, Fang Liu, Haibin Zhu, Xiaoyi Chen, Yuezhu Wang, Lanjuan Li, Karen E Nelson, Yaxian Xia, Charlie Xiang

**Affiliations:** 1 State Key Laboratory for Diagnosis and Treatment of Infectious Diseases, the First Affiliated Hospital, College of Medicine, Zhejiang University, Hangzhou, Zhejiang, 310003, China; 2 Zhejiang-California International Nanosystems Institute (ZCNI), Zhejiang University, Hangzhou, Zhejiang, 310029, China; 3 Department of Obstetrics and Gynecology, the First Affiliated Hospital, College of Medicine, Zhejiang University, Hangzhou, Zhejiang, 310003, China; 4 Chinese National Human Genome Center at Shanghai, Shanghai, 201203, China; 5 J. Craig Venter Institute, 9704 Medical Center Drive, Rockville, Maryland 20850, USA

## Abstract

**Background:**

Bacterial vaginosis (BV) is an ecological disorder of the vaginal microbiota that affects millions of women annually, and is associated with numerous adverse health outcomes including pre-term birth and the acquisition of sexually transmitted infections. However, little is known about the overall structure and composition of vaginal microbial communities; most of the earlier studies focused on predominant vaginal bacteria in the process of BV. In the present study, the diversity and richness of vaginal microbiota in 50 BV positive and 50 healthy women from China were investigated using culture-independent PCR-denaturing gradient gel electrophoresis (DGGE) and barcoded 454 pyrosequencing methods, and validated by quantitative PCR.

**Results:**

Our data demonstrated that there was a profound shift in the absolute and relative abundances of bacterial species present in the vagina when comparing populations associated with healthy and diseased conditions. In spite of significant interpersonal variations, the diversity of vaginal microbiota in the two groups could be clearly divided into two clusters. A total of 246,359 high quality pyrosequencing reads was obtained for evaluating bacterial diversity and 24,298 unique sequences represented all phylotypes. The most predominant phyla of bacteria identified in the vagina belonged to *Firmicutes*, *Bacteroidetes*, *Actinobacteria *and *Fusobacteria*. The higher number of phylotypes in BV positive women over healthy is consistent with the results of previous studies and a large number of low-abundance taxa which were missed in previous studies were revealed. Although no single bacterium could be identified as a specific marker for healthy over diseased conditions, three phyla - *Bacteroidetes*, *Actinobacteria *and *Fusobacteria*, and eight genera including *Gardnerella*, *Atopobium*, *Megasphaera*, *Eggerthella*, *Aerococcus*, *Leptotrichia*/*Sneathia*, *Prevotella *and *Papillibacter *were strongly associated with BV (*p *< 0.05). These genera are potentially excellent markers and could be used as targets for clinical BV diagnosis by molecular approaches.

**Conclusions:**

The data presented here have clearly profiled the overall structure of vaginal communities and clearly demonstrated that BV is associated with a dramatic increase in the taxonomic richness and diversity of vaginal microbiota. The study also provides the most comprehensive picture of the vaginal community structure and the bacterial ecosystem, and significantly contributes to the current understanding of the etiology of BV.

## Background

An enormous number of microorganisms, the vast majority of which are bacterial species, are known to colonize and form complex communities, or microbiota, at various sites within and on the human body [1,2]. Microbial cells that thrive on and within the human body are approximately 10 times more numerous than our own cells and contain, in aggregate, about 100 times more genes, leading to the suggestion that humans and our microbial symbionts be considered "supraorganisms" [3]. A growing body of evidence suggests that the composition and function of the microbiota in different human body habitats plays a vital role in human development, physiology, immunity, and nutrition [1,4-8]. As one of the important human- microbial habitats, the vagina harbors different species of bacteria in very large numbers that are known to have important effects on health [9]. Many of these bacteria such as hydrogen peroxide- and lactic acid- producing *Lactobacillus *spp. are not simply passive or transient colonizers, but rather appear to be adapted to the specific environment of the vagina [10-12]. These resident species effectively constitute an ecological guild - a group of species that have similar requirements and play a similar role within a community - and maintaining high numbers of these populations is a hallmark of healthy conditions [13]. The dramatic changes in the types and relative proportions of the microbial species in the vagina could lead to a diseased state [14]. One important component of the Human Microbiome Project (HMP), states that it is necessary to explore the bacterial diversity of vagina in health and disease and to understand whether changes in the vaginal microbiome can be correlated with changes in human health [2].

BV is the most prevalent lower genital tract infection in women of reproductive age throughout the world [15-18]. It affects millions of women annually [19] and is strongly associated with several adverse health outcomes, including preterm labor and delivery [20,21], pelvic inflammatory disease [22], postpartum and postabortal endometritis [23], and increased susceptibility to infection with various pathogens, such as *Neisseria gonorrhoeae*, *Trichomonas vaginalis, Chlamydia trachomatis*, *Candida*, and even HPV, HSV-2, and HIV-1 [24-30]. Abnormal vaginal discharge may be the only symptom of BV, and many affected women are asymptomatic [31]. Zhou *et al*. have suggested that a certain number of women without any symptoms do have vaginal communities that resemble BV [13]. Previous studies have demonstrated that BV is a polymicrobial syndrome, characterized by a shift in vaginal microbiota from a predominant population of *Lactobacillus *spp. [14,32] to their gradual or total replacement with anaerobes such as *Gardnerella vaginalis *[33], *Morbiluncus *spp. [34], *Prevotella *spp. [14], *Mycoplasma hominis *[21] and the recently identified metronidazole resistant *Atopobium vaginae *[35]. These species are however also found in subjects who do not suffer from BV with low copy numbers and thus cannot be used as a specific marker for disease.

Because of our inability to cultivate most of the microbial species that reside in the vagina, we have not fully understood the taxonomic composition of the vaginal microbiota, its community structure, and ultimately, its function. With the advent of new molecular techniques, we have been able to investigate bacterial diversity in different microhabitats using molecular fingerprinting methods and sequence analysis of microbial small subunit ribosomal (r) RNA genes (16S rRNA) and other universal targets (such as *cpn60*) [13,36,37]. Among these molecular fingerprinting methods, PCR-DGGE represents a rapid and reliable technique to identify the predominant microbiota in various ecological niches [38,39]. Sequencing of 16S rRNA genes from different samples by constructing clone libraries, (typically at most a few thousand clones from a low number of individuals), has revolutionized our understanding of microbial systematics and diversity [40,41]. However, this cloning and sequencing method identifies only the predominant microorganisms in a sample. Detection of low-abundance taxa requires analysis of datasets that are orders of magnitude larger than those currently available [42]. The recently available high-throughput 454 pyrosequencing now allows for very in-depth sequencing and analysis of microbial community composition, and also allow for a dramatic increase in throughput via parallel in-depth analysis of a large number of samples with limited sample processing and lower costs [43-45].This technique has been successfully used in various ecosystems including deep mines [46], soil [47], fermented seafood [48], skin [49], chronic wounds [50], and oral microbiota [42].

In order to better estimate the diversity of the vaginal community of the healthy and BV positive women, and to identify the key population changes relevant to BV development, we first utilized PCR-DGGE with broad range primers that correspond to the bacterial 16S rRNA hypervariable V3 region to investigate the predominant vaginal microbiota in these populations. We then used massively parallel pyrosequencing, combined with a DNA barcoding, to characterize the overall structure and composition of the vaginal bacterial community in 50 BV and 50 CN (non-infected healthy women; CN) individuals in China. We also quantified the abundance of total *Bacteria *and bacterial subgroups that associated significantly with BV using quantitative PCR (qPCR). Our data provides a more comprehensive picture over current knowledge of the community structure of the vaginal bacterial ecosystem. These results also help to define the potential pathogenic populations in BV and provide new insights into the etiology and treatment of the disease.

## Results

### PCR-DGGE analysis of vaginal bacterial communities

**Figure 1:**
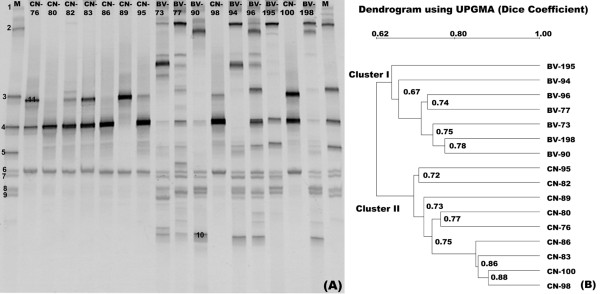
**Figure 1 PCR-DGGE analysis of the predominant bacterial communities in vaginal swabs from bacterial vaginosis (BV group) and healthy women (CN group).** (A) Each lane of the PCR-DGGE gel represented one subject which was selected in its group at random. M represents a marker constructed in this study with the identified bands to facilitate the interpretation of the figure. Bands: 1: Uncultured Sneathia sp.; 2: Fusobacterium nucleatum subsp. nucleatum ATCC 25586; 3: Clostridium thermocellum ATCC 27405; 4: Lactobacillus iners; 5: Clostridium acetobutylicum; 6: Lactobacillus iners; 7: Clostridium thermocellum ATCC 27405; 8: Atopobium vaginae; 9: uncultured Eggerthella sp.; 10: uncultured Megasphaera sp.; 11: Lactobacillus crispatus. (B) Dendrogram of the DGGE profiles shown in panel A.

PCR-DGGE is a useful tool to examine microbial diversity and community structure in specific microhabitats and has been widely applied for comparative analysis of parallel samples. The differences of DGGE profiles in the same community DNA samples might be caused by different primer sets selected targeting the different hypervariable regions of the 16S rRNA genes. The hypervariable region(s) chosen for amplification can influence the PCR-DGGE profiles and diversity indices produced from community DNA samples, and even subtle differences in primer sequences can result in substantially different profiles and downstream assessments of microbial diversity. By comparing different hypervariable regions of 16S rRNA genes for PCR-DGGE, Yu and Morrison (2004) have shown that the DGGE profiles of the V3 region were the most reliable [51]. As shown in Figure 1 A, the PCR-DGGE profiles of BV and CN revealed significant differences in the overall structure and composition of the vaginal community by targeting the V3 region of 16S rDNA. Bacterial diversity was higher in the BV group than that in the CN group. DGGE profiles were significantly different from one another and varied with the participants. Figure 1 B depicts the results of Ward's analysis in which the Dice coefficient for measuring similarity in banding patterns was applied. The BV and CN groups displayed a statistically significant clustering of profiles, cluster Ⅰ (BV group) and cluster II (CN group). 11 dominant fragments that could represent the pattern of the DGGE profiles were excised from the DGGE lanes, reamplified, sequenced and identified by BLAST with the 16S rRNA V3 region sequences. *Lactobacillus *was the predominant genus in the CN group and bacterial diversity of the BV group was far more complex and was dominated by *A. vaginae*, uncultured *Sneathia sp*., *Fusobacterium nucleatum subsp*., uncultured *Eggerthella sp*., uncultured *Megasphaera sp*., *Clostridium acetobutylicum *and *Clostridium thermocellum*. From these results, we propose that PCR-DGGE analysis could be used to monitor the dramatic shift of bacterial transition and routinely defined BV in laboratory.

### Sequence analysis by pyrosequencing

From 50 BV and 50 CN individuals, more than 321,400 PCR amplicons of the V3 hyper-variable regions of the 16S rRNA gene were sequenced, of which a total of 246,359 pyrosequencing tags passed quality control, and were included in our data analysis. Specifically, we obtained 90,227 sequences from the BV group and 156,132 sequences from the CN group respectively. The average length of the sequences was 145 bp after trimming the primers and the average number of sequence reads was 2,464 per sample. The total number of unique sequences from the two groups was 24,298 and represented all phylotypes. It was unexpected that we obtained more sequences from CN samples than from BV samples (*p *< 0.05, data not shown) because the same amount of bacterial DNA was used for preparing the library for pyrosequencing. It can be assumed that the biased ratio of the two groups was not only because of an inefficiency in the emPCR technique for different species of vaginal bacteria, but also because of the impurities or unknown compounds disturbing the accurate measurement of DNA amount [48,52].

### The diversity of vaginal microbiota in BV and CN individuals

**Figure 2:**
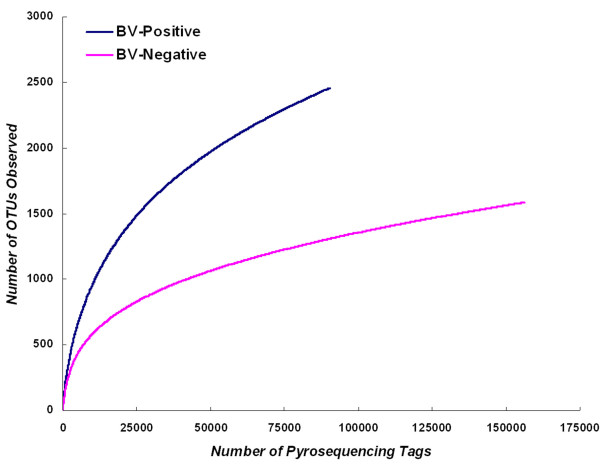
**Rarefaction curves were used to estimate richness (in this case the number of taxa at a 3% dissimilarity level) among BV-positive and BV-negative groups**. The vertical axis shows the number of OTUs that would be expected to be found after sampling the number of tags or sequences shown on the horizontal axis.

**Figure 3:**
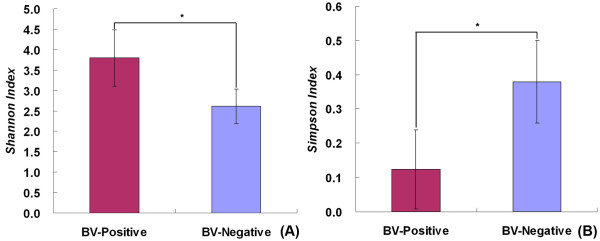
**Shannon index and Simpson index were used to estimate diversity (i.e., a combined assessment of the number of 1% dissimilar bacterial taxa and their abundance) among the eight groups**. Data shown as mean with SEM. There were significant differences between BV-positive and BV-negative groups by parametric ANOVA.

**Figure 4:**
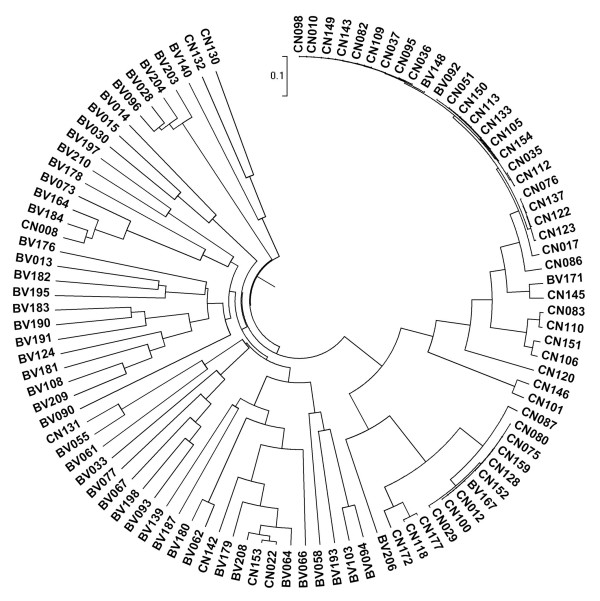
**Differentiation in vaginal bacterial communities from 100 individual samples of BV-positive and BV-negative groups (interpersonal variations)**. Community differentiation was measured by using the unweighted UniFrac algorithm; the scale bar indicates the distance between clusters in UniFrac units. All of the branch nodes shown here were found to be significant (*p <*0.001), indicating that BV-positive and BV-negative harbored distinct bacterial communities.

The numbers of species detected in a sample, or the numbers of organisms discerned at any given phylogenetic level, are strongly affected by the number of sequences analyzed. Rarefaction analysis provides a powerful method for evaluating the diversity and richness of different microhabitats in the human body. Rarefaction curves were generated for unique, 1%, 2%, 3%, 5% and 10% sequence dissimilarities as described previously [47]. As shown in Figure 2, we found that there was much more richness in bacterial diversity in BV individuals than in CN individuals at the 3% dissimilarity level. The number of OTUs continued to increase at the 3% or 5% dissimilarity level in the BV group, however, the rarefaction curve in the CN group almost reached the saturation level, which indicated that additional sampling was needed to determine the true microbial diversity in the BV vaginal community. Based upon the literature, the nonparametric ACE and Chao1 estimators are correlated positively with the number of sequences analyzed [53] and overestimate the number of species while the rarefaction estimator underestimates the number of species at the 0% dissimilarity level. However, the rarefaction estimator could accurately predict the number of species at the 3% dissimilarity level and the number of genera at the 5% dissimilarity level. For the two groups of vaginal communities analyzed at the 3% dissimilarity level, the number of OTUs detected was close to the total number of OTUs estimated by Chao1 and ACE diversity indices, presenting additional evidence that the natural communities were well covered during sequencing (Table 1). Good's coverage was more than 99.0% for all sequences in the two groups, indicating that less than one additional phylotype would be expected for every 100 additional sequenced reads. This level of coverage indicated that the 16S rRNA sequences identified in these groups represent the majority of bacterial sequences present in the samples under study. With the unique sequences analyzed, the species diversity was evaluated with Shannon Index (2.6133 in the CN group Vs. 3.7965 in the BV group) and Simpson Index (0.3793 in the CN group Vs. 0.1240 in the BV group). So, the overall levels of bacterial diversity was significantly different between the BV group and the CN group (*p *< 0.05 for the two groups, parametric ANOVA for Shannon and Simpson index) (Figure 3), and bacterial communities were significantly different between individuals from the two groups. The vaginal community in the BV group (Evenness = 0.3327) was more even than in the CN group (Evenness = 0.2185) and indicated that bacterial community in the BV group had greater species diversity. All these parameters corroborated each other and confirmed clearly that the vaginal community was more diverse in BV positive women than in BV negative women. The samples in each group or in an individual sample were divided into two clusters based on UniFrac metrics (Figure 4), although there were few BV samples which clustered in the CN group and vice versa. Although more subclusters existed in the BV group, we found that samples in the BV group formed a large cluster that was very distinct from the CN group, which was similar to the PCR-DGGE pattern, indicating that they harbored quite different microbial communities.

**Table 1:** Comparison of phylotype coverage and diversity estimation of the 16S rRNA gene libraries at the 3% dissimilarity from the pyrosequencing analysis

*Group*	*Reads*	***OTUs***^**1**^	***Good***^**2**^	*ACE*	***95% C.I***.	*Chao*	***95% C.I***.	*Shannon*	***Evenness***^**3**^	*Simpson*
BV-Negative	156,132	1,584	0.996	2,857	(2,714, 3,000)	2,500	(2,375, 2,625)	2.6133	0.2185	0.3793
BV-Positive	90,227	2,455	0.990	4,009	(3,808, 4,209)	3,694	(3,509, 3,879)	3.7965	0.3327	0.1240

^**1**^The operational taxonomic units (OTUs) were defined with 3% dissimilarity level.

^**2**^The coverage percentage (Good), richness estimators (ACE and Chao1) and diversity indices (Shannon and Simpson) were calculated using Good's method (Good, 1953) and the MOTHUR program.

^**3**^The Shannon index of evenness was calculated with the formula E = H/ln(S), where H is the Shannon diversity index and S is the total number of sequences in that group.

**Figure 5:**
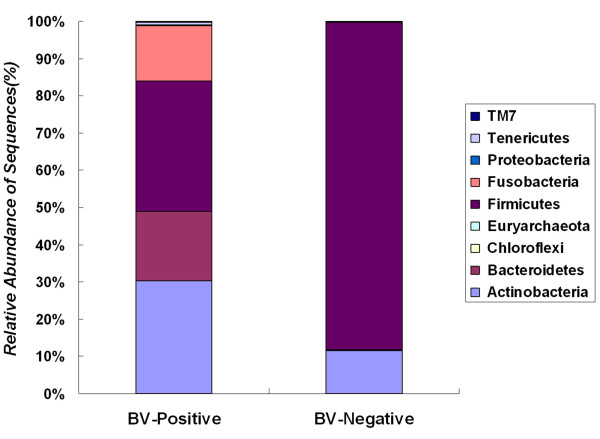
**The relative abundance of vaginal bacterial V3 tags obtained by pyrosequencing from BV-positive and BV-negative individuals, by phylum**. Phylogenetic classification for the pyrosequencing analysis obtained from Ribosomal Database Project Classifier analyses. The phyla of *Firmicutes*, *Bacteroidetes*, *Actinobacteria*, *Fusobacteria *were significant differences between BV-positive and BV-negative groups by parametric ANOVA (*p <*0.000).

**Figure 6:**
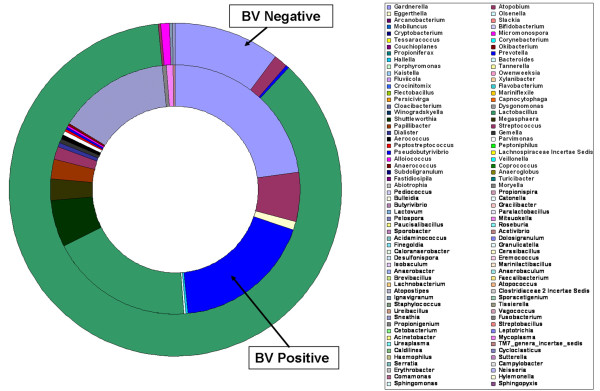
**The relative abundance of vaginal bacterial V3 tags obtained by pyrosequencing from BV-positive and BV-negative individuals, by genus and profiled the overall structure of vaginal communities**.

The phylogenetic classification of sequences from the vagina by phylum is summarized in Figure 5. The composition and relative abundance of the vaginal microbiota by phylum might be not help one to understand the etiology of BV. However, it did reveal the overall structure of the vaginal microbiota. Eight phyla were found in the vaginal microbiota in women with or without BV. Our data showed that the vast majority of sequences belonged to one of the four major phyla: *Bacteroidetes*, *Firmicutes*, *Actinobacteria *and *Fusobacteria*. Of these major phyla, *Firmicutes *was the most dominant phyla in the vaginal microbiota of healthy subjects, while bacteria belonging to *Firmicutes*, *Bacteroidetes*, *Actinobacteria *and *Fusobacteria *constituted the complex vaginal microbiota in the BV group. The remaining bacteria belonged to the phyla *Chloroflexi*, *Tenericutes*, *Proteobacteria *and candidate division TM7 (around 0.1-1.0% of total sequences). The composition of the vaginal microbiota at the phylum level was significantly associated with BV by SPSS with One-Way ANOVA (*p <*0.001). At the genus level, sequences from the two combined groups represented 116 different genera, with 99 different genera in the BV group and 58 different genera in the CN group respectively (the taxonomy of the vaginal bacterial communities with RDP classifier showed details in Additional file 1, Table S1). Many genera found in the vaginal community were unexpected, and our results suggest that this is one of the most comprehensive investigations of vaginal microbiota conducted to date (Figure 6). Eleven genera (*Lactobacillus*, *Gardnerella*, *Atopobium*, *Megasphaera*, *Eggerthella*, *Aerococcus*, *Alloiococcus*, *Streptococcus*, *Leptotrichia*/*Sneathia*, *Prevotella *and *Papillibacter*) constituted more than 95% of the vaginal microbiota. Not surprisingly, our study showed that the genus *Lactobacillus *constituted the major proportion of vaginal microbiota in healthy women. This confirmed findings from previous studies [13,14].

**Figure 7:**
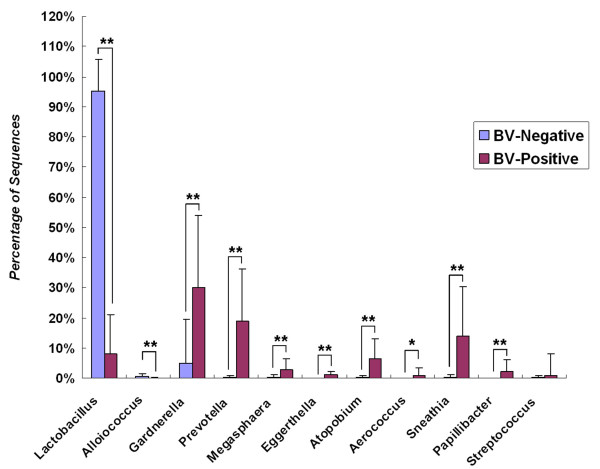
**Predominant genera detected in vagina from BV-positive and BV-negative individuals**. Among these predominant genera, *Lactobacillus*, *Gardnerella*, *Atopobium*, *Alloiococcus*, *Sneathia*, *Prevotella*, *Papillibacter*, *Megasphaera*, *Eggerthella*, *Aerococcus *were associated with BV significantly (* *p *< 0.05, ** *p *< 0.01). Except for *Lactobacillus *and *Alloiococcus*, which were detected at a higher level, other genera were more abundant in BV-positive individuals.

**Figure 8:**
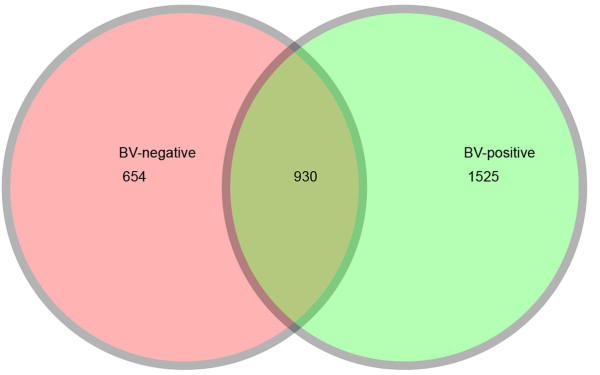
**Venn diagrams for overlap between BV-positive observed OTUs versus BV-negative observed OTUs**. The Venn diagrams show the overlap in all OTUs calculated at the 3% dissimilarity level. The number of species in group BV-positive is 2,455. The number of species in group BV-negative is 1,584. The number of species shared between groups BV-positive and BV-negative is 930. Percentage of species that are shared in groups BV-positive and BV-negative is 29.91%.

There was a significant difference between the BV and CN group in all genera obtained from the vagina. The abundance of *Lactobacillus*, *Alloiococcus*, *Gardnerella*, *Atopobium*, *Megasphaera*, *Eggerthella*, *Aerococcus*, *Leptotrichia*/*Sneathia*, *Prevotella *and *Papillibacter *was significantly different between the two groups (*p *< 0.05) (Figure 7). This indicated that the composition and abundance of the vaginal bacterial communities including these genera played an important role in BV. At the species level, defined as OTUs at the 3% dissimilarity level, around 1,500 species and about 2,500 species were found in the CN and BV group, respectively. While at a more conservative level, defined as OTUs at the 5% dissimilarity level, about 550 phylotypes in the CN group and about 1,000 phylotypes in the BV group were found. The two groups also shared a great degree of community similarity in the vagina at the 3% dissimilarity level--about 30% of the total phylotypes were present in both groups (Figure 8). Our Venn diagrams agree with previous findings that some genera in vaginal communities were not specific for BV, but also existed in the healthy women.

### Total bacteria and species-specific qPCR

Our qPCR results supported the pyrosequencing results in terms of which species were associated with BV, although the relative abundance was not in concordance (Additional file 2, Table S2). Consistent with previous findings, we found that *L. iners *was a major component of the vaginal microbiota in healthy women and decreased markedly in BV subjects. *Gardnerella*, *Atopobium*, *Megasphaera*, *Eggerthella*, *Leptotrichia*/*Sneathia *and *Prevotella *were more common and present at a higher copy numbers in the BV group, although there were significant interpersonal variations (data not shown). Our qPCR revealed the bacterial subgroups that associated significantly with BV and verified the results of PCR-DGGE and pyrosequencing data. Our quantitative studies of the bacterial species in the vaginal communities demonstrated one common finding: increased numbers of these bacteria were found during the advent of BV. Compared with vaginal microbiota in healthy women, the relative abundance or copy numbers of these bacteria combined with each other was associated with BV significantly and could be potentially used as a molecular marker of microbiota shift in vagina and as a target for diagnosis of BV.

### Comparison of pyrosequencing with PCR-DGGE analysis

The bacterial diversity of vaginal microbiota was analyzed by PCR-DGGE fingerprinting and barcoded pyrosequencing. These phylotypes detected in the vagina matched each other with two molecular analytical methods. PCR-DGGE fingerprinting, which is a conventional molecular ecological approach, only detected the predominant microbiota (*Lactobacillus*, *A. vaginae*, uncultured *Sneathia *sp., *F. nucleatum *subsp., uncultured *Eggerthella *sp., and uncultured *Megasphaera *sp.) in the vagina, and these phylotypes were also abundant in the 454 pyrosequencing reads. However, the bacterial communities determined with the pyrosequencing analysis were more diverse than those communities determined with the PCR-DGGE analysis, as there were more phylotypes revealed with the pyrosequencing analysis than with PCR-DGGE fingerprinting. As we could not excise and sequence all bands in the DGGE profiles, several dominant bands were missed, of which might contain some predominant bacteria such as *G. vaginalis *in the vaginal communities. Pyrosequencing provided a high-throughput approach to analyze the 16S rRNA gene sequences and explore bacterial diversity in different microhabitats deeply, which can compensate for the disadvantage with the PCR-DGGE method in detecting minor populations in microbiota. As only predominant microbiota could be detected by PCR-DGGE, band richness could not reveal the overall extent of bacterial diversity in the vagina. With clustering analysis using the UniFrac algorithm, we found similar cluster profiles in the two groups. The samples in the BV and CN group were divided into two clusters respectively (Figure 1B and Figure 4). To analyze bacterial diversity, PCR-DGGE fingerprinting and high-throughput pyrosequencing were two important and useful methods, which could corroborate one another.

## Discussion

An understanding of the composition and richness of the vaginal microbial ecosystem in relation to vaginal health is essential for comprehensively understanding the etiology of vaginal diseases, and for the prevention and treatment of these diseases. We analyzed the vaginal communities of healthy participants and women with clinically defined BV which was diagnosed by Amsel criteria and clearly confirmed by Nugent criteria with Nugent score ≥ 7. Indeed, few of these subjects were asymptomatic BV, where the shift of vaginal microbiota has occurred but the symptoms are absent. However, both the Gram staining and PCR-DGGE verified our diagnosis. Although there are several previous studies that focus on the vaginal microbiota in health and disease or in different races, molecular analysis such as the broad-range PCR assays, terminal restriction fragment length polymorphisms (T-RFLPs) and PCR-clone library analysis still cannot demonstrate the overall structure and composition of vaginal microbiota comprehensively [13,14,54]. In fact, the depth of the bacterial diversity in a specific microhabitat is strongly influenced by the total number of sequences that are used for analysis [47]. These previous studies obtained only hundreds of reads for each sample which severely limited the depth of understanding of the overall structure of vaginal microbiota. However, pyrosequencing of the 16S rRNA gene amplicons for microbial community profiling can, for equivalent costs, yield more than two orders of magnitude more sensitivity than traditional PCR cloning and Sanger sequencing [55]. In our study, multiplex barcoded pyrosequencing analysis enabled us to analyze an increased number of samples at a time, to obtain more reads in a single run, to monitor the sequencing error rate, and to perform in-depth analyses for studies of comparative microbial ecology. Although the pyrosequencing read lengths of the sequences were significantly short compared with the sequences obtained with traditional Sanger sequencing methods (600-800bp), these short sequences (about 145bp in our study) provided not only excellent coverage but also excellent recovery for classification at the genus level [56]. In addition, the 145bp in the highly variable region V3 of the 16S rRNA gene had good discerning power (provided that a 1bp difference in the 16S rRNA gene sequences differentiated the reads by 0.55% for bacteria) and were long enough to be sufficient for assigning the taxa [48]. Engelbrektson *et al*. (2010) have shown that for a given number of reads, shorter 16S rRNA gene amplicons yield greater species richness than do longer amplicons. Approximately 100bp amplicons produced significantly higher estimates of richness than 400bp or 1,000bp products did [57]. All indicated that multiplex parallel pyrosequencing offered a highly automated, rapid, economical and accurate method for the analysis of bacterial diversity. Our study represents one of the most extensive investigations of bacterial diversity in the vagina.

With 454 pyrosequencing analysis, we found that the vaginal bacterial diversity in participants with BV was remarkably high and much greater than previously reported with culture-independent approaches such as PCR-DGGE and cloning and sequencing [13,14,37,39,58] and similar with the deep-sequencing techniques by Spear *et al*. (2008) [59]. The higher number of phylotypes in BV positive women over healthy was consistent with the results of previous studies, and most of which were previously uncultivated species and undiscovered novel phylotypes in the vagina. Compared with other molecular methods for microbiota diversity analysis such as cloning and sequencing approaches, we observed higher bacterial diversity by high-throughput parallel pyrosequencing analysis (Good's coverage more than 99.0%) [13,54,60]. The rarefaction curves were still not saturated at the 3% dissimilarity level even if more than 150,000 sequences in the CN group were retrieved, which indicated that more sequencing effort should be undertaken to obtain the saturation of the curves. However, the Good's coverage of more than 99.0% of our study showed that we had obtained almost all bacterial species in the vaginal communities. Recently, a computational model revealed that more than 9 million unique genes were likely to be present in the human gut bacterial community. It was far more rich than had been expected [61]. Based on more pyrosequencing reads and good coverage, that's why the depth of the vaginal communities in our study was superior to those previous studies [13,14,54,58,59]. Other diversity indices shown in Table 1, such as Shannon index, Chao1 and ACE, were also higher in the BV group than in the CN group at the same dissimilarity level. Furthermore, for each sample analyzed in our study, these diversity indices showed significantly interpersonal variations even in the same group (data not shown), similar to that observed for the gastrointestinal tract and human skin [40,62,63]. The variation might be due to hormone levels, personal hygiene behavior and even diet [64-66]. Although there were pronounced interpersonal variations in the bacterial community composition of the vagina, the participants also shared a great degree of community similarity in each group and the overall structure of the vaginal bacterial communities in the two groups was obviously divided into two clusters.

Our study represents one of the most extensive examinations of bacterial diversity in the vagina. We demonstrated in this research that BV was associated with compositional changes in the vaginal microbiota mostly apparent at high taxonomic level (phylum) and even genus level. From the eight phyla that we observed in the vaginal ecosystem, *Firmicutes *constituted the vast majority of vaginal microbiota in healthy women, while *Bacteroidetes*, *Actinobacteria *and *Fusobacteria *were strongly associated with BV. It is not surprising that the predominant bacterial populations in the healthy vagina contained lactic acid bacteria (mainly the genus of *Lactobacillus*) [67] because these genera can maintain a low vaginal pH through their metabolic activity and thereby play a role in colonization resistance providing protection against invasion by overt pathogens or against overgrowth and dominance by potentially pathogenic species among the normal microbiota [68-70]. However, the genera of *Alloiococcus *(member of the lactic acid bacteria), which was previously an unrecognized common member of the healthy vaginal ecosystem, was detected more frequently as normal microbiota in the healthy women in our study. Our data firstly indicated that the members of *Alloiococcus *along with members of the *Lactobacillus *might contribute to maintaining the balance of the healthy vaginal ecosystem. Extensive diversity within the *Lactobacillus *species complex of the vagina has been observed in previous studies. With species-specific qPCR, we also found that *L. iners *was one of the most abundant vaginal *Lactobacillus *species in healthy women, while about 100 to 1000-fold copies declined or were even absent in the subjects with BV. Research has shown that *L. iners *is a dominant part of the vaginal microbiota when in a transitional stage between abnormal and normal health, either because of treatment or because of physiological changes such as varying estrogen levels [71,72]. As a result, *L. iners *could be considered as even more typical for normal vaginal microbiota and be a sensitive marker of changes in the vaginal microbiota. Other species of *Lactobacillus *such as *L. crispatus *and *L. jensenii *were also commonly detected *Lactobacillus *species in the vaginal microbiota but at a lower relative abundance. Previously, *L. crispatus *has been suggested to be more often linked with health and *L. iners *with disease [72]. However, no clear quantitative support was found for this claim in our study.

The onset of BV is marked by a decline in the *Lactobacillus *species and other facultative or anaerobic species as the vaginal microbial ecosystem changes from eubiosis to dysbiosis [73]. In our study, eight BV-related genera, *i.e*., *Gardnerella*, *Atopobium*, *Megasphaera*, *Eggerthella*, *Aerococcus*, *Leptotrichia*/*Sneathia*, *Prevotella *and *Papillibacter*, were detected at higher prevalence and higher relative abundance in women with BV. These genera included pathogenic species that participated in BV progression. Previous studies have shown that *G. vaginalis *(belonging to *Actinobacteria*), was found in most subjects with BV and showed higher sensitivity for the diagnosis of BV [14,33,74], but our data also found this species in subjects with low abundance level who did not have BV. This suggests that this species cannot be used as a specific marker for this disease. The difference might be associated with the depth of the pyrosequencing method. Despite its non-specific discrimination between BV and CN, we did observe that it was one of most important species in the vaginal communities that associate with BV. Another genus in *Actinobacteria*, *Atopobium*, which plays an important role in the development of BV and treatment failure, is highly abundant in the vaginal microbiota of BV [35]. Our pyrosequencing study showed that *Atopobium *is present in a significant proportion (84%) of women clinically diagnosed as having BV, but in only 22% women in CN group (*p *< 0.05). Although *Atopobium *was also a lactic acid bacterium, its cellular morphology and role was distinctly different from that of *Lactobacillus *[75]. With this aspect, Bradshaw *et al*. (2006) suggested that *Atopobium *was even more specific than *G. vaginalis *in BV (77% and 35%, respectively) [76]. Our results also confirmed this implication. *Eggerthella*, like *A. vaginae *in this phylum, was strongly correlated with BV. Tamrakar *et al*. (2007) reported that the presence of *Eggerthella *was an independent risk factor of BV scores (Nugent score ≥ 7) [77]. In our study, we highlighted this genus in the vaginal bacterial community in the process of BV. Many studies have shown that *Mobiluncus *(belonging to Actinomycetaceae in the phylum of *Actinobacteria*) was found in vaginal bacterial communities only when BV was present, and had a high-level resistance to metronidazole [78]. However, we obtained only low abundance of this genus in our study, which was consistent with a previous study [60]. Other members of the *Firmicutes*, *Megasphaera *(especially *Megasphaera *typeⅠ), *Aerococcus *and *Papillibacter*, were also associated with BV significantly, although these genera could not be detected in all BV samples. However, the clinical significance of these genera in the vaginal ecosystem is still unknown. Previous work has shown that *Megasphaera *typeⅠ appeared to have a stronger association with BV than type Ⅱ [79]. We also found that *Megasphaera *typeⅠwas common in BV patients and those two unfamiliar genera of *Aerococcus *and *Papillibacter *in vaginal bacterial communities were obviously correlated with BV for the first time. *Prevotella *(belonging to *Bacteroidetes*), one of the recently identified predominant microbiota in the complex vaginal communities of BV subjects, was significantly associated with BV in our qPCR and pyrosequencing studies (*p *< 0.05). *Prevotella *was dramatically higher (7 orders of magnitude) in almost all patients with the clinically defined BV in our qPCR experiment. Our data showed that *Prevotella *spp. were more common and abundant than other BV-associated genera in the vagina. Ammonia flow from *Prevotella *to *G. vaginalis *has been demonstrated and a commensal relationship proposed [80]. It might be implicated that *Prevotella *showed a synergistic effect with *G. vaginalis *and aggravated the process of BV. The combination of bacterium-specific PCR assays for *Prevotella *and *G. vaginalis *might improve sensitivity or specificity for the diagnosis of BV. *Leptotrichia*/*Sneathia *(belonging to *Fusobacteria*), which was also a lactic acid-producer, was strongly associated with BV in our study. However, there was little known about the ecology of this genus. Fredricks *et al*. (2005) showed that detection of *Leptotrichia *species was very specific for BV by bacterium-specific PCR assays [14]. Our observations in qPCR and pyrosequencing studies showed that the prevalence of *Leptotrichia*/*Sneathia *in BV patients was higher than in CN subjects. It could be a new marker of BV in the pathogenic vaginal communities like others mentioned above. Compared with the ten predominant genera, other bacteria identified from the complex vaginal bacterial communities were detected at low relative abundance. Some have been detected in the vagina before, but most of them were first found in our study.

However, there were also some limitations to our study. First, there was no analysis of Nugent score and pH in relation to the change of vaginal community structure and composition as participants diagnosed as BV in our study were almost with Nugent score ≥ 7 and a pH of vaginal discharge ≥ 4.5. For such an analysis it would be necessary to explore the relationship between intermediate vaginal microbiota (4 ≤ Nugent score < 7) and asymptomatic BV. Second, we did not explore the dynamics of vaginal bacterial communities in the process of BV and the bacterial diversity for re-establishment of vaginal microbiota after effective treatment by the high-throughput pyrosequencing. Further studies of the vaginal microbiota-host interplay, especially those bacteria found in our study with low relative abundance and its influence on local vaginal immunity, will be necessary in order to understand extensively the ecological role of the complex vaginal bacterial communities in the BV process. This new insight into the overall structure of vaginal community in BV may provide fundamental information for future investigations.

## Conclusions

Our results elucidated that vaginal microbiota are more diverse in BV participants than we expected before. This provides novel insights into the vaginal microbiota in the etiology of BV and confirms that the barcoded pyrosequencing approach can be a powerful tool for characterizing the microbiota in vaginal ecosystems compared with classical molecular ecological approaches, such as PCR-DGGE. The study represents one of the most extensive examinations of bacterial diversity in the vagina. We observed that at a high taxonomic level, the phylum of *Bacteroidetes*, *Actinobacteria *and *Fusobacteria *were significantly associated with BV. Although no single bacterium can be identified as uniquely associated with BV, our data indicated that the vaginal communities including *Gardnerella*, *Atopobium*, *Megasphaera*, *Eggerthella*, *Aerococcus*, *Leptotrichia*/*Sneathia*, *Prevotella *and *Papillibacter *were clearly associated with BV. These genera in the vaginal communities were potentially excellent markers of BV and could be used as targets for BV diagnosis by molecular approaches alone or in combination. Our results have provided a comprehensive picture of our current knowledge of the community structure of the vaginal bacterial ecosystem and have significantly increased current understanding of the etiology of BV. Continuing exploration of vaginal bacterial communities and host interplay in different healthy and diseased states will shed light on the BV susceptibility and provide new insights for treatment.

## Methods

### Subject Selection

One hundred women with regular menstrual cycles (24-35 days) aged 19 to 51 years, including 50 BV positive women (BV group, aged 33.3 ± 9.1) and 50 healthy control women (CN group, aged 32.0 ± 8.1), who came to the Department of Obstetrics and Gynecology of the First Affiliated Hospital, College of Medicine, Zhejiang University for routine gynecology examination from October 2008 to May 2009, were recruited for this study. Informed written consent was obtained from all participants prior to enrollment, with approval of the ethical committee of the First Affiliated Hospital, College of Medicine, Zhejiang University, Zhejiang province, China. Individuals who participated in this study were examined by two gynecologists. BV status was assessed using Amsel clinical criteria for all subjects [81] and confirmed using Gram stain criteria (Nugent scores) [82]. Only participants with Nugent score ≥ 7 were selected for the following analysis. Any participant having any of the following exclusion criteria was excluded from participation: <18 years of age, pregnancy, diabetes mellitus, the use of antibiotics or vaginal antimicrobials (orally or by topical application in vulvar/vaginal area) in the previous month, menstruation, presence of an intrauterine device, vaginal intercourse within the latest 3 days, known active infection due to *Chlamydia*, yeast, *N. gonorrhoeae*, or *T. vaginalis*, clinically apparent herpes simplex infection, or defined diagnosed HPV, HSV-2, or HIV-1 infection. (The clinical data for each participant were shown in Additional file 3, Table S3). The participants who met three or more of the following criteria were clinically diagnosed as BV: homogenous vaginal discharge, >20% clue cells on wet mount, elevated pH (≥4.5) of vaginal discharge, and release of a fishy amine odor upon addition of 10% potassium hydroxide solution to vaginal fluid ("whiff" test) [81]. Then based on the criteria for BV assessment developed by Nugent *et al*. [82], participants with the Gram stain score of ≥7 were finally confirmed as BV. Participants without these changes were defined as the healthy control group.

### Sample Collection and Preparation

When women underwent genital examination, 2 swabs from each woman were taken near the mid-vagina using a sterile swab, packaged and placed in ice packs. (Kim *et al*. (2009) [83] have revealed that the vaginal microbiota is not homogenous throughout the vaginal tract but differs significantly within an individual with regard to anatomical site and sampling method used.) The first swab was rolled onto a slide for Gram staining; the second vaginal swab was used for bacterial genomic DNA extraction. The vaginal swabs for bacterial genomic DNA extraction were transferred to the laboratory immediately in an ice-box, and stored at -80°C after preparation within 15 min for further analysis.

### Total bacterial genomic DNA extraction

The bacterial cells retrieved on swabs were submerged in 1 ml of sterile normal saline (prepared with RNase free H_2_O, pH 7.0) and vigorously agitated to dislodge cells. The cells were pelleted by centrifugation (Thermo Electron Corporation, Boston, MA, USA) at full speed (≥ 10,000 g) for 10 min, washed by re-suspending cells in sterile normal saline and centrifuged at full speed for 5 min. Then bacterial DNA was extracted from the vaginal swabs using QIAamp DNA Mini Kit (QIAGEN, Hilden, Germany) according to manufacturer's instructions with minor modification. Briefly, the bacterial pellet was suspended in 180 μl of lysis buffer (buffer ATL) and homogenized by vortexing. A total of 20 μl of a proteinase K solution (20 mg/ml) and 100 mg of zirconium beads (0.1 mm) were then added. The mixtures were agitated in a mini bead beater (FastPrep, Thermo Electron Corporation, USA) three times, 40 s each time, and incubated at 56°C for approximately 40 min. 200 μl of the second lysis buffer (buffer AL) provided in the kit was added, and the sample was incubated at 70°C for 10 min. Next, 200 μl of ethanol was added; this mixture was then loaded on the QIAamp spin column and centrifuged at 8,000 g for 1 min. The QIAamp spin column was placed in a new 2 ml collection microtube, and the containing filtrate was discarded. The column material was washed with 500 μl buffer AW1 and with 500 μl buffer AW2 provided in the kit. Finally, the DNA was eluted with 20 μl of distilled water (2 × 10 μl). The concentration of extracted DNA was determined by using a NanoDrop ND-1000 spectrophotometer (Thermo Electron Corporation, USA); its integrity and size were checked by 1.0% agarose gel electrophoresis containing 0.5 mg/ml ethidium bromide. All DNA was stored at -20°C before further analysis.

### PCR-DGGE analysis

Universal bacterial primers 341F and 534R for the V3 regions of 16S rRNA genes were used to amplify approximately 200 bp, based on positions 341 to 534 of the *Escherichia coli *16S rRNA gene, as described by Muyzer *et al*. (1993) [38,51]. The reaction conditions were those as described previously by Li *et al*. (2008) [41] with minor modifications. The DGGE analysis (with 35% to 50% gradient) and the sequence analysis of the excised DGGE bands was performed as described [41]. The similarities of PCR-DGGE DNA profiles were analyzed with Quantity One^® ^1-D Analysis software (version 4. 6.2; Bio-Rad Laboratory, Hercules, CA, USA). A similarity matrix was constructed using Dice's similarity coefficient. A dendrogram was constructed by the unweighted pair group method, using arithmetic averages (UPGMA) (details in Additional file 4, Supplementary Information).

### 454 pyrosequencing and data analysis

PCR amplification of the 16S rRNA gene hypervariable V3-region was performed with universal bacterial primers (It must be noted that 454 adaptor sequences and barcodes are not shown here): 341F (5'- ATTACCGCGGCTGCTGG -3') and 534R (5'-CCTACGGGAGGCAGCAG -3') (details in Additional file 4, Supplementary Information) as described [48]. Amplicon pyrosequencing was performed with standard 454/Roche GS-FLX protocols [84]. After pyrosequencing, all reads were screened and filtered for quality and length using customized Perl scripts written by ourselves. Raw sequences were processed and analyzed following the procedure described previously [43]. Approximately 23.3% of the total raw pyrosequencing reads was not passed quality control. Sequences were assigned to samples by examining the 8-bp barcode (Additional file 5, Table S4) [85]. The qualified 16S rRNA gene fragments were processed as previously described [86]. OTUs and OTU rarefaction curves were created by aligning unique tag sequences and used to determine richness and diversity indexes (Shannon Weaver and Simpson diversity indices), ACE, Chao1, Good's coverage at each dissimilarity level using MOTHUR (version 1.5.0) http://schloss.micro.umass.edu/[87]. Read level taxonomic assignments were performed using the Ribosomal Database Project (RDP) Classifier program http://rdp.cme.msu.edu/[88] with an 80% bootstrap score. Community comparative analysis was performed using the web-based service UniFrac [89]. The neighbor-joining tree was constructed using the MEGA 4.0 program based on the Jukes-Cantor model and used for UniFrac analysis. The statistical significance of differences in microbial community composition, and Shannon and Simpson index between sample categories was determined by SPSS with One-Way ANOVA (details in Additional file 4, Supplementary Information).

### Validation of the relative abundance of vaginal microbiota with qPCR

To estimate the accurate copy numbers of total *Bacteria *and bacterial subgroups in the samples, and to validate the relative abundance of genus-specific bacteria as determined by 454 pyrosequencing. 16S rRNA gene-targeted qPCR (species-specific primers in Additional file 6, Table S5) was performed with a Power SYBR Green PCR Master Mix (Takara, Dalian, China) on an ABI 7900 Real-time PCR instrument according to the manufacturer's instructions (Applied Biosystems, Foster city, CA). Details of the standards and PCR conditions are provided in Additional file 4, Supplementary Information.

## Authors' contributions

ZXL conducted the experimental design, sample collection, PCR-DGGE, real-time PCR, barcodes design, analyzed results and wrote the paper. JMK carried out experimental design, samples collection, barcodes design, PCR amplification and analyzed results. FL, HBZ and YXX participated in sample collection and partial data analysis. XYC carried out quantitative real-time PCR and data analysis. YZW carried out partial data analysis. LJL, KEN, YXX and CX conceived of the study, and participated in its design and coordination and helped to draft the manuscript. All authors read and approved the final manuscript.

## Supplementary Material

Additional file 1 **Table S1. The taxonomy of vaginal bacterial communities with RDP Classifier**. Taxonomic assignments at the genus level were made with a bootstrap confidence range at ≥ 80% using the RDP Classifier. Click here for file

Additional file 2 **Table S2. The abundance of vaginal bacteria relative to total *Bacteria *gene copy number by species-specific qPCR**. Comparisons of the relative abundance of the specific bacteria in the vagina between women with BV and women without BV were calculated with independent-samples T-tests (SPSS Data Analysis Program version 16.0, SPSS Inc, Chicago, IL) and were considered statistically significant if *p *< 0.05. These qPCR results supported the pyrosequencing results in terms of which species were associated with BV, although the relative abundance was not in concordance. Click here for file

Additional file 3 **Table S3. The Clinical Data for Each Participant**. 100 subjects (50 women with BV and 50 women without BV) that participated in our study were diagnosed with Amsel clinical criteria and Nugent criteria. Click here for file

Additional file 4 **Supplementary Information**. Supplemental materials and methods used in our study. Click here for file

Additional file 5 **Table S4. List of the 100 8-bp barcodes used to tag each PCR product analyzed as part of the study**. Table of 8-bp barcodes used to identify each sample from the sample pool. Click here for file

Additional file 6 **Table S5. Species-specific primer sets for detection of vaginal bacteria by qPCR**. Table of primers used in this study to carry out real-time PCR analysis of total bacteria and 10 bacterial species in the vagina. Click here for file
