# Supplementary material for: Molecular analysis of the diversity of vaginal microbiota associated with bacterial vaginosis
Source: BMC Genomics. 2010 Sep 7;11:488. doi: 10.1186/1471-2164-11-488 (PMC2996984; doi:10.1186/1471-2164-11-488)
Supplement: Additional file 2 — Table S2. The abundance of vaginal bacteria relative to total Bacteria gene copy number by species-specific qPCR. Comparisons of the relative abundance of the specific bacteria in the vagina between women with BV and women without BV were calculated with independent-samples T-tests (SPSS Data Analysis Program version 16.0, SPSS Inc, Chicago, IL) and were considered statistically significant if p < 0.05. These qPCR results supported the pyrosequencing results in terms of which species were associated with BV, although the relative abundance was not in concordance. [file 1471-2164-11-488-S2.DOC]

**Table S2** The abundance of vaginal bacteria relative to total *Bacteria* gene copy number by species-specific qPCR

| Species-Specific qPCR | Group | | | | *p* value**^1^** |
| --- | --- | --- | --- | --- | --- |
|  | BV-Negative | | BV-Positive | |  |
|  | Mean | Std. Deviation | Mean | Std. Deviation |  |
| *Lactobacillus* genus | 75.55% | 22.70% | 10.74% | 16.59% | 0.0000 |
| *L. crispatus***^2^** | 3.45% | 10.90% | 0.00% | 0.00% | 0.2839 |
| *L. iners***^2^** | 61.59% | 30.85% | 1.68% | 2.93% | 0.0000 |
| *L. jensenii***^2^** | 1.25% | 2.08% | 0.74% | 2.56% | 0.6157 |
| *G. vaginalis* | 0.00% | 0.00% | 2.96% | 4.14% | 0.0356 |
| *A. vaginae* | 0.00% | 0.00% | 7.71% | 8.97% | 0.0136 |
| *Eggerthella* | 0.00% | 0.00% | 0.52% | 0.42% | 0.0008 |
| *Megasphaera* type 1 | 0.00% | 0.00% | 2.58% | 4.79% | 0.1055 |
| *Leptotrichia/Sneathia* | 0.00% | 0.00% | 5.86% | 5.16% | 0.0019 |
| *Prevotella* | 0.01% | 0.00% | 47.52% | 0.29% | 0.0001 |

**^1^** Comparisons between women with BV and women without BV were calculated with independent-samples T-tests (SPSS Data Analysis Program version 16.0, SPSS Inc, Chicago, IL) and were considered statistically significant if *p* < 0.05.

**^2^** The relative abundance of *L. crispatus*, *L. iners* and *L. jensenii* was compared to the copy number of *Lactobacillus* genus.
