# Supplementary material for: Molecular analysis of the diversity of vaginal microbiota associated with bacterial vaginosis
Source: BMC Genomics. 2010 Sep 7;11:488. doi: 10.1186/1471-2164-11-488 (PMC2996984; doi:10.1186/1471-2164-11-488)
Supplement: Additional file 4 — Supplementary Information. Supplemental materials and methods used in our study. [file 1471-2164-11-488-S4.DOC]

**Supplementary Information**

**PCR amplification of DNA for DGGE and identification**

The V3 regions of the 16S rRNA genes were amplified by universal bacterial primers (341F 5’- GTATTACCGCGGCTGCTGG -3’, 534R 5’-CGCCCGCCGCGCGCGGCGGGCGGGG

CGGGGGCACGGGGGGACTCCTACGGGAGGCAGCAG -3’). For DGGE analysis, a 40-bp GC clamp (5’-CGC CCG CCG CGC GCG GCG GGC GGG GCG GGG GCA CGG GGG G) was attached to the 5’ end of reserve primers. The 16S rRNA-V3 PCR amplification used the hot-start touchdown protocol described by Muyzer *et al.* (1993) [1], and the reaction mixture contained 1.25 unit of Hot Start Taq polymerase (Takara, Dalian, China), 1× PCR buffer (2.5 mM MgCl_2_ included), 3 pmol of each primer, 200 mM each deoxynucleoside triphosphate (dNTP) and 20 ng of extracted bacterial DNA in a total volume of 50 μl. The thermal cycling program consisted of the following time and temperature profile: 95°C for 5 min; 20 cycles of touchdown PCR: denaturizing at 95°C for 30 s, annealing at 65°C for 30 s which was decreased by 0.5°C every second cycle, and extending at 72°C for 30 s; then additional 5 cycles with annealing temperature at 55°C for 30 s were performed; followed by final extension at 72°C for 7 min. To minimize heteroduplex formation and single-stranded DNA (ssDNA) contamination during PCR amplification that might cause sequence heterogeneity in a single DGGE band, an additional 5 cycles of reconditioning PCR and PCR products were purified by electrophoresis on a 1% agarose gel and eluted with QIAquick Gel Extraction Kit (QIAGEN) before DGGE analysis. All PCRs were performed in a thermocycler PCR system (DNA Engine Tetrad 2, Bio-Rad, Hemel Hempstead, Herts, UK). Samples (5 μl) of the amplified products (approximately 200 bp) were checked by electrophoresis on 1% (wt/vol) agarose gel and visualized by ethidium bromide staining, and the concentrations were measured by using a NanoDrop ND-1000 spectrophotometer (Thermo Electron Corporation). All amplified products were stored at -20°C before DGGE analysis.

**DGGE analysis**

Parallel DGGE analysis was performed using the D-Code universal mutation detection system apparatus (Bio-Rad, Hercules, CA) with 16-cm by 16-cm by 1-mm gels according to the manufacturer's protocol. The sequence-specific separation of the PCR fragments was obtained in 8% (wt/vol) polyacrylamide (acrylamide-N, *N’* bisacrylamide; 37.5:1 [wt/vol]) gels in 1× TAE buffer (40 mM Tris, 20 mM glacial acetic acid, 1 mM EDTA, pH 8.0). The denaturing gels contained a 35% to 50% gradient of urea and formamide increasing in the direction of electrophoresis. A 100% denaturing solution contained 40% (vol/vol) formamide and 7 M urea. A stacking gel containing 8% (wt/vol) polyacrylamide was applied onto the denaturing gel. A volume of 13 to 16 μl of PCR samples was loaded onto the stacking gel. Electrophoresis was conducted at a constant voltage of 200 V and a temperature of 60°C for approximately 4 h. Following electrophoresis, the gel was stained by SYBR green I (Amresco, Ohio, USA) and photographed with UVI gel documentation system (UVItec). Each set of samples was independently PCR amplified and analyzed by DGGE twice to confirm the reproducibility of banding profiles.

Fragments of interest were excised from denaturing gradient gels with a sterile scalpel and placed into a single Eppendorf tube. Gel pieces were washed once in 1 x PCR buffer and incubated in 20 μl of the same buffer overnight at 4°C. Five microliters of the buffer solution was used as template for PCR re-amplification with universal bacterial primers, as described above for DGGE, but without GC clamps. PCR products were excised from 1.0% agarose gel and purified with QIAquick Gel Extraction Kit (QIAGEN), then ligated with pGEM-T Easy Vector (Promega), transformed into competent *Escherichia coli* DH5α cells. The positive clones were verified and sequenced (Invitrogen, Shanghai, China). The sequences of excised DGGE bands were submitted to RDP II release10 database to determine their closest isolate relatives with length <1 200 bp. Sequences similarity searches were used to assign each clone to major bacterial phylotypes.

**Analysis of DGGE profiles**

The digitized gel images were analyzed using Quantity One® 1-D Analysis software (version 4. 6.2; Bio-Rad Laboratory, Hercules, CA, USA). The software was used to detect bands by normalizing against the total intensity data for each lane. Bands with a minimum density of 5% were detected in each lane and bands were matched using a match tolerance of 2%. Bands occupying the same position in the different lanes of the gels were identified and the DGGE fingerprint lane contained 249 variables. A similarity matrix was constructed using Dice's similarity coefficient. This is defined as [2j / (a + b)] x 100, where j is the number of bands in common between two lanes, and (a+b) is the total band number of both lanes. Dendrograms were constructed by the unweighted pair group method, using arithmetic averages (UPGMA).

**PCR Amplification and Sample Pooling for 454 pyrosequencing**

PCR amplification of the 16S rRNA gene hypervariable V3-region was performed with universal bacterial primer which corresponded to positions 341 to 534 in *Escherichia coli*. Amplicon pyrosequencing was performed with standard 454/Roche GS-FLX Titanium protocols. To pool and sort multiple samples in a single 454 GS-FLX run, we designed unique barcode of 8 nucleotides to identify each sample (designated by NNNNNNNN; see Additional file 5, Table S4). We used a set of 8-bp barcodes designed according to Fierer *et al.* [2-4]. The main criterion of these barcodes is that the adjoining nucleotides must be different because the single nucleotide repeats are the main source of errors in pyrosequencing technology. The resulting forward primer was a fusion of the 454 life science adaptor A, the barcode, and 341F (5’-gcctccctcgcgccatcag-NNNNNNNN-ATTACCGCGGC TGCTGG -3’). And the resulting reverse primer was a fusion of the 454 life science adaptor B, the same barcode with forward primer, and 534R (5’- gccttgccagcccgctcag -NNNNNNNN- CCTACGGGAGGCAGCAG -3’). The PCR amplicon library was created for each individual DNA sample. The amplification mix contained 1.25 U of Hot Start Taq polymerase (Takara, Dalian, China), 1 x PCR buffer (2.5 mM MgCl_2_ included), 3 pmol of each primer, 200 mM each deoxynucleoside triphosphate (dNTP) and 1 μl of extracted bacterial DNA in a total volume of 50 μl. Samples were initially denatured at 94 °C for 5 min, then amplified by using 30 cycles of 94 °C for 30 s, 55 °C for 30 s, and 72 °C for 30 s. A final extension of 7 min at 72 °C was added at the end of the program to ensure complete amplification of the target region. Negative controls (both no-template and template from unused swabs) were included in all steps of the process to check for primer or sample DNA contamination. Before pooling these 100 samples, PCR products were purified by electrophoresis on a 1% agarose gel and eluted with QIAquick Gel Extraction Kit (QIAGEN). The concentration of each PCR product was measured by using a NanoDrop ND-1000 spectrophotometer (Thermo Electron Corporation) three times and then quantified by using on-chip gel electrophoresis with Agilent 2100 BioAnalyzer (Agilent Technologies, Santa Clara, CA, USA) and DNA LabChip Kit 7500. Individual amplicon libraries were pooled in equimolar amounts, and subjected to emulsion PCR, and generated amplicon libraries and sequenced unidirectionally in the reverse direction (B-adaptor) by means of the Genome Sequencer FLX (GS-FLX) system (Roche, Basel, Switzerland) at 454 Life Sciences. Because samples were pooled by equal mass, variation in the number of sequences recovered from each sample likely reflects slight biases in PCR efficiency among primer barcodes.

**Data analyses for 454 pyrosequencing reads**

**Sequence processing pipeline**

Initially, all pyrosequencing reads were screened and filtered for quality and length of the sequences using customized Perl scripts. Raw sequences were processed and analyzed following the procedure described previously [4]. Sequences were included in the subsequent analysis, only if the sequences met all four of the following criteria: (1) the sequence carries the correct barcode and exact match to the primer in at least one end; (2) the sequence carries the correct primer sequence in the other end, even though the barcode absent; (3) the sequence has a length of longer than 160 nucleotides (excluding barcode and primer A sequences) [5]; and (4) the sequence without any ambiguous bases (Ns). Because all of the samples are pooled into a single sequencing reaction, we incorporate the barcodes to allow reads from each individual sample to be identified. In this way, we could analyze the sequences from each sample separately. Sequencing reads were derived directly from FLX sequencing run output files. This resulting multi-FASTA file contained 246,359 total high-quality reads. Based upon the individual sample barcode sequence, those sequences specific for each sample were extracted from the multi-FASTA file into individual FASTA files. The sequences were then relabeled according to denote the original sample. In order to facilitate the subsequent pipeline pyrosequencing analysis, the script then trimmed off the forward primer sequence and barcode sequence after alignment and oriented the remaining sequence such that all sequences begin with the 5’ end according to standard sense strand conventions. We included only sequences with the forward primer motif to ensure that the highly informative V3 region was available for taxonomic assignment.

**Phylogenetic assignment, alignment and clustering of 16S rRNA gene fragments**

The qualified 16S rRNA gene fragments were phylogenetically assigned according to their best matches to sequences based upon BLASTn using WND-BLAST [6] and a curated 16S database derived from high quality 16S sequences obtained from RDPII database [7]. Phylogenetic assignments were also evaluated using the Nearest Alignment Space Termination (NAST, <http://greengenes.lbl.gov/NAST>) database [8]. Multiple sequence alignment was done using a newly update version of DOTUR, called MOTHUR (version 1.5.0) (<http://schloss.micro.umass.edu/mothur/Main_Page>) [9] [align.seqs](http://schloss.micro.umass.edu/mothur/Align.seqs) command. MOTHUR is a new computational toolbox for describing and comparing microbial communities, which are designed to be a platform that will enable to align their 16S rRNA gene sequences, calculate pairwise distances, and analyze the resulting distance matrices. With the MOTHUR filter.seqs command, we will remove any vertical gaps from the alignment. Based on the alignment, a distance matrix at a given % dissimilarity was constructed using with MOTHUR dist.seqs command. The proportion of OTUs shared among the communities was determined using MOTHUR, which uses the .list output files from MOTHUR as input and determines the fraction of OTUs shared by communities as a function of genetic distance. These pairwise distances served as input to MOTHUR for clustering the sequences into OTUs of defined sequence similarity that ranged from unique to 3% dissimilarity. A distance matrix was then generated using the MOTHUR tree.shared command. These clusters served as OTUs for generating predictive rarefaction models and for making calculations with the richness (diversity) indexes ACE and Chao1 [10] in MOTHUR. These programs were run on a SUSE Linux Enterprise 10 machine, 24 quad core 48 processors at 3.0 Ghz with 128 GB of RAM.

**Statistical data analysis of OTU richness: rarefaction, Chao 1 and ACE**

To estimate species richness and diversity, taxonomy-independent methods were used. Clustering was done with a given % dissimilarity for inclusion into an OTU and was performed on alignments of sequences from individual participant. The matrices were used to define operational taxonomic units with 1% dissimilarity for determination of the coverage percentage by Good’s method. The species richness and relative abundance of species (Evenness) was estimated by further sampling-based (rarefaction) analyses of OTU data and of calculated Shannon and Simpson diversity indices. These clusters served as OTUs for generating rarefaction curves and for making calculations with the richness and diversity indexes, abundance based coverage estimator (ACE), bias-corrected Chao1 richness estimator, in MOTHUR at each dissimilarity level. Shannon index characterize diversity based on the number of species present (species richness). The Shannon index of evenness was calculated with the formula E = H/ln(S), where H is the Shannon diversity index and S is the total number of sequences in that group. This metric is insensitive to the taxa richness and ranges from 0 to 1, with 0 representing complete dominance and 1 representing an evenly structured community. Good’s coverage percentage was calculated as [1/ (n/N)]/100, where n represents the number of single-member phylotypes and N represents the number of sequences. The resulting tables of OTU clusters versus dataset and primer were the source data for the Venn diagrams. We plotted our Venn diagrams using the Venn Diagram Plotter program written by Littlefield and Monroe at the Department of Energy, PNNL, Richland, VA. Taxonomy-based analyses were performed by assigning taxonomic status to each sequence using the Naïve Bayesian CLASSIFIER program of the Michigan State University Center for Microbial Ecology Ribosomal Database Project (RDP) database (<http://rdp.cme.msu.edu/>) [11] with an 80% bootstrap score. Sequences were aligned using Infernal Aligner both from individual participant and then as pooled sequences from all participants of a single group. Cluster analysis was performed using the complete linkage clustering algorithm available through the Pyrosequencing pipeline of the Ribosomal Database Project [12]. The neighbor-joining tree was constructed using the MEGA 4.0 program based on the Jukes-Cantor model and used for UniFrac analysis. Statistical analyses for Shannon and Simpson index were performed using SPSS Data Analysis Program version 12.0 (SPSS Inc, Chicago, IL) with One-Way ANOVA.

**Real-time qPCR for vaginal microbiota**

To estimate the accurate copy numbers of bacteria in vagina samples and validate the relative abundance of bacteria in genus determined by 454 pyrosequencing, 16S rRNA gene-targeted quantitative PCR (qPCR) was performed with a Power SYBR Green PCR Master Mix (Takara, Dalian, China) on an ABI 7900 Real-time PCR instrument according to the manufacturer’s instructions (Applied Biosystems, Foster city, CA). Species-specific primer sets were chosen to quantify total bacteria, *Lactobacillus genus*, *L. iners*, *L. crispatus*, *L. jensenii*, *Gardnerella vaginalis*, *Atopobium vaginae*, *Eggerthella* sp., *Megasphaera type*Ⅰsp., *Leptotrichia*/*Sneathia* sp. and *Prevotella* sp. (Additional file 6, Table S5). For each primer set, a constructed plasmid was chosen to create a 10-log-fold standard curve for direct quantification of all samples. With the exception of total domain *Bacteria* and *Lactobacillus* *genus*, all standard curve genes were amplified from the vaginal samples, constructed plasmids, sequenced and confirmed the source of target organisms by BLAST in Genebank. For total domain *Bacteria* and *Lactobacillus genus*, *Escherichia coli* ATCC 25922 and *Lactobacillus casei* ATCC 27139 was used to create the plasmid standards, respectively. For each, the product was cloned into pMD18-T vector using the Simple TA Cloning Kit (Takara, Dalian, China) following the manufacturer’s procedure. Purified insert-containing plasmids were quantified using a NanoDrop ND-1000 spectrophotometer (Thermo Electron Corporation), and taking into account the size of the product insert, the number of target gene copies was calculated from the mass of DNA. Tenfold serial dilutions ranging from 1 x 10^9^ to 1 gene copies were included on each 96-well plate. Each subject’s extracted DNA was subjected to a human β-Globin PCR to ensure that amplifiable DNA was successfully extracted from the sample and to monitor for PCR inhibitors with the same protocol listed for bacterial PCR [13]. Each qPCR contained 12.5 μL of 2 x Takara Perfect Real Time master mix, 10.9 μL of water, 0.3 μL of a 10 μM F/R primer mix, and 1 μL of extracted bacterial genomic DNA. Cycling conditions: 95 °C for 3 min; 40 repeats of the following steps: 94 °C for 30 s, 30 s annealing at different temperature, and 72°C for 30 s. At each cycle, accumulation of PCR products was detected by monitoring the increase in fluorescence of the reporter dye, dsDNA-binding SYBR Green. Following amplification, melting temperature analysis of PCR products was performed to determine the specificity of the PCR. Melting curves were obtained from 55 °C to 90 °C, with continuous fluorescence measurements taken at every 1 °C increase in temperature. Data analysis was conducted with Sequence Detection Software version 1.6.3, supplied by Applied Biosystems. All reactions were carried out in triplicate and a nontemplate control was performed in every analysis. In addition, the abundance of each group relative to total domain *Bacteria* gene copy number was calculated for each replicate, and the mean, standard deviation and statistical significance were determined. Comparisons between women with BV and women without BV were calculated with unpaired t-tests (SPSS Data Analysis Program version 16.0, SPSS Inc, Chicago, IL) and were considered statistically significant if *p* < 0.05.

**References**

1. Muyzer G, de Waal EC, Uitterlinden AG: **Profiling of complex microbial populations by denaturing gradient gel electrophoresis analysis of polymerase chain reaction-amplified genes coding for 16S rRNA.** *Appl Environ Microbiol* 1993 **59:** 695-700.

2. Fierer N, Hamady M, Lauber CL, Knight R: **The influence of sex, handedness, and washing on the diversity of hand surface bacteria.** *Proc Natl Acad Sci U S A* 2008 **105:** 17994-17999.

3. Parameswaran P, Jalili R, Tao L, Shokralla S, Gharizadeh B, Ronaghi M, Fire AZ: **A pyrosequencing-tailored nucleotide barcode design unveils opportunities for large-scale sample multiplexing.** *Nucleic Acids Res* 2007 **35:** e130.

4. Hamady M, Walker JJ, Harris JK, Gold NJ, Knight R: **Error-correcting barcoded primers for pyrosequencing hundreds of samples in multiplex.** *Nat Methods* 2008 **5:** 235-237.

5. Roh SW, Kim KH, Nam YD, Chang HW, Park EJ, Bae JW: **Investigation of archaeal and bacterial diversity in fermented seafood using barcoded pyrosequencing.** *ISME J* 2010 **4:** 1-16.

6. Dowd SE, Zaragoza J, Rodriguez JR, Oliver MJ, Payton PR: **Windows .NET Network Distributed Basic Local Alignment Search Toolkit (W.ND-BLAST).** *BMC Bioinformatics* 2005 **6:** 93.

7. Cole JR, Chai B, Farris RJ, Wang Q, Kulam-Syed-Mohideen AS, McGarrell DM, Bandela AM, Cardenas E, Garrity GM, Tiedje JM: **The ribosomal database project (RDP-II): introducing myRDP space and quality controlled public data.** *Nucleic Acids Res* 2007 **35:** D169-172.

8. DeSantis TZ, Hugenholtz P, Larsen N, Rojas M, Brodie EL, Keller K, Huber T, Dalevi D, Hu P, Andersen GL: **Greengenes, a chimera-checked 16S rRNA gene database and workbench compatible with ARB.** *Appl Environ Microbiol* 2006 **72:** 5069-5072.

9. Schloss PD, Westcott SL, Ryabin T, Hall JR, Hartmann M, Hollister EB, Lesniewski RA, Oakley BB, Parks DH, Robinson CJ, Sahl JW, Stres B, Thallinger GG, Van Horn DJ, Weber CF: **Introducing mothur: open-source, platform-independent, community-supported software for describing and comparing microbial communities.** *Appl Environ Microbiol* 2009 **75:** 7537-7541.

10. Chao A, Bunge J: **Estimating the number of species in a stochastic abundance model.** *Biometrics* 2002 **58:** 531-539.

11. Wang Q, Garrity GM, Tiedje JM, Cole JR: **Naive Bayesian classifier for rapid assignment of rRNA sequences into the new bacterial taxonomy.** *Appl Environ Microbiol* 2007 **73:** 5261-5267.

12. Cole JR, Wang Q, Cardenas E, Fish J, Chai B, Farris RJ, Kulam-Syed-Mohideen AS, McGarrell DM, Marsh T, Garrity GM, Tiedje JM: **The Ribosomal Database Project: improved alignments and new tools for rRNA analysis.** *Nucleic Acids Res* 2009 **37:** D141-145.

13. Fredricks DN, Relman DA: **Paraffin removal from tissue sections for digestion and PCR analysis.** *Biotechniques* 1999 **26:** 198-200.
