# Supplementary material for: Molecular analysis of the diversity of vaginal microbiota associated with bacterial vaginosis
Source: BMC Genomics. 2010 Sep 7;11:488. doi: 10.1186/1471-2164-11-488 (PMC2996984; doi:10.1186/1471-2164-11-488)
Supplement: Additional file 5 — Table S4. List of the 100 8-bp barcodes used to tag each PCR product analyzed as part of the study. Table of 8-bp barcodes used to identify each sample from the sample pool. [file 1471-2164-11-488-S5.DOC]

**Table S4 List of the 100 8-bp barcodes used to tag each PCR product analyzed as part of the study**

| **ACAGCTAT** | **ACTACATG** | **ACGATAGC** | **CAGATACA** |
| --- | --- | --- | --- |
| **ACGTGACT** | **ACAGCAGT** | **ACTGTACG** | **CAGTGTCA** |
| **CATGTCTG** | **ACGTACTC** | **AGATACAC** | **AGTGCGAT** |
| **CATAGTAG** | **ACTCGCAC** | **AGCGCTGA** | **ATACTCAC** |
| **ATCATCAT** | **AGAGAGCA** | **ACACATGT** | **ATCGCTCG** |
| **ATCGAGCT** | **AGCATATG** | **ACATGATC** | **ATGCAGCT** |
| **TGTACTCG** | **AGTCTACA** | **ACGCATCT** | **ATACTGTG** |
| **TGATCGAG** | **ACGCTGCA** | **ACTGTCGA** | **CACGCACT** |
| **CTACGATG** | **ACAGCTAG** | **AGCGTAGC** | **CAGATCGC** |
| **TCGACGAC** | **ACGTGTCT** | **ACACGAGC** | **CATACGAG** |
| **ATCGCACG** | **ACGTCTGT** | **ACATGTCA** | **AGTGCATG** |
| **ACACTGAT** | **AGAGCATG** | **ACGCGATA** | **ATAGCTCG** |
| **ACGAGACG** | **AGCGATAC** | **ACTACGTG** | **ATCGTACA** |
| **ACGCTCAT** | **AGCTGTGA** | **ACTGTGAC** | **CATCACGC** |
| **ACTCACGC** | **ACTCAGTC** | **AGATCTCT** | **CACGTCGA** |
| **AGACTGTC** | **ACGACGTC** | **AGCTATCG** | **CAGCACTA** |
| **AGCACGAG** | **ACGTGAGA** | **AGTCTATA** | **CATAGACG** |
| **ACAGACTA** | **ACTGACAG** | **ACACGCTG** | **AGTGTCAC** |
| **ACGAGCGA** | **AGCGACTG** | **ACATACAG** | **ATACGCGA** |
| **ACGCATCG** | **AGTACGCT** | **ACTAGTAG** | **ATCTACTA** |
| **AGCATGAC** | **ACTCGTGA** | **AGATGTAC** | **ATGCGTAG** |
| **AGCTGTGC** | **ACGAGTGC** | **AGCTCGAT** | **CATAGCGA** |
| **ACAGCAGA** | **ACTGATCG** | **ACGCTATC** | **AGTGTACG** |
| **ACGCTGAG** | **AGAGTCGT** | **ACTATAGT** | **ATATCGCT** |
| **ACTCGATA** | **AGCGAGCT** | **AGATCACG** | **ATCTCTCG** |
