# Supplementary material for: Molecular analysis of the diversity of vaginal microbiota associated with bacterial vaginosis
Source: BMC Genomics. 2010 Sep 7;11:488. doi: 10.1186/1471-2164-11-488 (PMC2996984; doi:10.1186/1471-2164-11-488)
Supplement: Additional file 6 — Table S5. Species-specific primer sets for detection of vaginal bacteria by qPCR. Table of primers used in this study to carry out real-time PCR analysis of total bacteria and 10 bacterial species in the vagina. [file 1471-2164-11-488-S6.DOC]

**Table S5** Species-specific primer sets for detection of vaginal bacteria by qPCR

| **PCR specificity** | **Primer** | **Sequence**  **(5'-3')** | **Annealing**  **Temperature** | **Amplicon**  **size(bp)** | **Reference** |
| --- | --- | --- | --- | --- | --- |
| **All bacteria** | **Bac27F** | **AGAGTTTGATCCTGGCTCAG** | **65** | **312** | [1] |
|  | **EUB338R-I** | **GCTGCCTCCCGTAGGAGT** |  |  |  |
| ***Lactobacillus*** | **Bact-0011** | **AGAGTTTGATYMTGGCTCAG** | **62** | **667** | [2] |
|  | **Lab-0677** | **CACCGCTACACATGGAG** |  |  |  |
| ***L. crispatus*** | **Lcris-F** | **AGCGAGCGGAACTAACAGATTTAC** | **65** | **154** | [3] |
|  | **Lcris-R** | **AGCTGATCATGCGATCTGCTT** |  |  |  |
| ***L. jensenii*** | **Ljens-F** | **AAGTCGAGCGAGCTTGCCTATAGA** | **60** | **162** | [4] |
|  | **Ljens-R** | **CTTCTTTCATGCGAAAGTAGC** |  |  |  |
| ***L. iners*** | **Liners-F** | **CTCTGCCTTGAAGATCGGAGTGC** | **65** | **155** | [4] |
|  | **Liners-R** | **ACAGTTGATAGGCATCATCTG** |  |  |  |
| ***G.*** ***vaginalis*** | **GV1-F** | **TTACTGGTGTATCACTGTAAGG** | **62** | **332** | [5] |
|  | **GV3-R** | **CCGTCACAGGCTGAACAGT** |  |  |  |
| ***A. vaginae*** | **AV-F** | **TAGGTCAGGAGTTAAATCTG** | **62** | **156** | [6] |
|  | **AV-R** | **TCATGGCCCAGAAGACCGCC** |  |  |  |
| ***Eggerthella*** | **Egger-621F** | **AACCTCGAGCCGGGTTCC** | **60** | **239** | [7] |
|  | **Egger-859R** | **TCGGCACGGAAGATGTAATCT** |  |  |  |
| ***Megasphaera type*Ⅰ** | **MegaE-456F** | **GATGCCAACAGTATCCGTCCG** | **64** | **212** | [7] |
|  | **MegaE-667R** | **CCTCTCCGACACTCAAGTTCGA** |  |  |  |
| ***Leptotrichia/Sneathia*** | **Lepto-395F** | **CAATTCTGTGTGTGTGAAGAAG** | **60** | **252** | [7] |
|  | **Lepto-646R** | **ACAGTTTTGTAGGCAAGCCTAT** |  |  |  |
| ***Prevotella*** | **Prevo-F** | **CCAGCCAAGTAGCGTGCA** | **60** | **151** | [8] |
|  | **Prevo-R** | **TGGACCTTCCGTATTACCGC** |  |  |  |
| **β-Globin** | **GH2O** | **GAAGAGCCAAGGACAGGTAC** | **60** | **270** | [7] |
|  | **PCO4** | **CAACTTCATCCACGTTCACC** |  |  |  |

**References**

1. Lipp JS, Morono Y, Inagaki F, Hinrichs KU: **Significant contribution of Archaea to extant biomass in marine subsurface sediments.** *Nature* 2008 **454:** 991-994.

2. Heilig HG, Zoetendal EG, Vaughan EE, Marteau P, Akkermans AD, de Vos WM: **Molecular diversity of Lactobacillus spp. and other lactic acid bacteria in the human intestine as determined by specific amplification of 16S ribosomal DNA.** *Appl Environ Microbiol* 2002 **68:** 114-123.

3. Byun R, Nadkarni MA, Chhour KL, Martin FE, Jacques NA, Hunter N: **Quantitative analysis of diverse Lactobacillus species present in advanced dental caries.** *J Clin Microbiol* 2004 **42:** 3128-3136.

4. Tamrakar R, Yamada T, Furuta I, Cho K, Morikawa M, Yamada H, Sakuragi N, Minakami H: **Association between Lactobacillus species and bacterial vaginosis-related bacteria, and bacterial vaginosis scores in pregnant Japanese women.** *BMC Infect Dis* 2007 **7:** 128.

5. Zariffard MR, Saifuddin M, Sha BE, Spear GT: **Detection of bacterial vaginosis-related organisms by real-time PCR for Lactobacilli, Gardnerella vaginalis and Mycoplasma hominis.** *FEMS Immunol Med Microbiol* 2002 **34:** 277-281.

6. Ferris MJ, Masztal A, Martin DH: **Use of species-directed 16S rRNA gene PCR primers for detection of Atopobium vaginae in patients with bacterial vaginosis.** *J Clin Microbiol* 2004 **42:** 5892-5894.

7. Fredricks DN, Fiedler TL, Marrazzo JM: **Molecular identification of bacteria associated with bacterial vaginosis.** *N Engl J Med* 2005 **353:** 1899-1911.

8. Martin FE, Nadkarni MA, Jacques NA, Hunter N: **Quantitative microbiological study of human carious dentine by culture and real-time PCR: association of anaerobes with histopathological changes in chronic pulpitis.** *J Clin Microbiol* 2002 **40:** 1698-1704.
